# The *Drosophila* PGC-1α Homolog *spargel* Modulates the Physiological Effects of Endurance Exercise

**DOI:** 10.1371/journal.pone.0031633

**Published:** 2012-02-13

**Authors:** Martin J. Tinkerhess, Lindsey Healy, Matthew Morgan, Alyson Sujkowski, Erin Matthys, Li Zheng, Robert J. Wessells

**Affiliations:** Department of Internal Medicine, Institute of Gerontology, University of Michigan Medical School, Ann Arbor, Michigan, United States of America; University of Washington, United States of America

## Abstract

Endurance exercise is an inexpensive intervention that is thought to provide substantial protection against several age-related pathologies, as well as inducing acute changes to endurance capacity and metabolism. Recently, it has been established that endurance exercise induces conserved alterations in physiological capacity in the invertebrate *Drosophila* model. If the genetic factors underlying these exercise-induced physiological alterations are widely conserved, then invertebrate genetic model systems will become a valuable tool for testing of genetic and pharmacological mimetics for endurance training. Here, we assess whether the *Drosophila* homolog of the vertebrate exercise response gene PGC-1α *spargel (srl)* is necessary or sufficient to induce exercise-dependent phenotypes. We find that reduction of *srl* expression levels acutely compromises negative geotaxis ability and reduces exercise-induced improvement in both negative geotaxis and time to exhaustion. Conversely, muscle/heart specific *srl* overexpression improves negative geotaxis and cardiac performance in unexercised flies. In addition, we find that *srl* overexpression mimics some, but not all, exercise-induced phenotypes, suggesting that other factors also act in parallel to *srl* to regulate exercise-induced physiological changes in muscle and heart.

## Introduction

In order to facilitate longitudinal studies across ages in model organisms, we have developed an endurance training paradigm in the short lived, genetically tractable *Drosophila* system. Trained flies exhibit several physiological phenotypes reminiscent of those seen in vertebrate models, including increased motor capacity, cardiac function, and mitochondrial enzyme activity [1]. However, in order to maximize the advantages of an invertebrate genetic model system, it is first important to establish that known genetic components of vertebrate exercise are, in fact, conserved in invertebrate models such as *Drosophila*.

The most well-studied genetic factor in the vertebrate response to endurance training is the transcriptional co-factor PGC-1α. Chronic training of mammals results in upregulation of PGC-1α transcript [2], while bouts of exercise acutely increase its nuclear localization [3]. Functionally relevant increases in PGC-1α expression following exercise have been observed in both adipose tissue [4] and muscle [5], [6]. Upregulation of PGC-1α increases mitochondrial biogenesis [7], [8], upregulates mitochondrial gene expression and upregulates fatty acid oxidation [9], [10]. Muscle-specific overexpression of PGC-1α in mice improves exercise ability and significantly increases aerobic capacity without altering whole-body energy expenditure, body weight, or fat mass. [11], [12]. By contrast, deletion of PGC-1α in mice results in reduced mitochondrial number, blunted postnatal muscle growth, reduced muscle performance and reduced exercise capacity [13]. The extent to which these phenotypes are necessary or sufficient components of broader physiological changes that are induced by endurance exercise is not yet fully understood.

A homolog of PGC-1α, *spargel (srl)* [14], has been identified in *Drosophila*. *spargel* plays a significant role in intestinal tissue homeostasis and, like its vertebrate counterpart, upregulates mitochondrial biogenesis and activity [14], [15]. The conservation of the molecular role of *spargel* suggests that its physiological role in response to endurance exercise may also be functionally conserved. Here, we examine whether high levels of *spargel* expression are required for exercise training to induce physiological changes. Additionally, we examine whether muscle-specific expression of *spargel* is sufficient to mimic the effects of exercise on several aspects of fly physiology, including negative geotaxis, cardiac function, time to exhaustion, basal activity levels and respiration.

## Results

### Respiration Rate

Endurance exercise is thought to increase mitochondrial biogenesis and activity in vertebrates [16], with PGC-1α as an important intermediate [17], [18]. Endurance exercise is also known to increase the activity of mitochondrial enzymes in *Drosophila* [1]. We measured whole-body CO_2_ production of flies with a hypomorphic mutation for *spargel* (*srl*), the *Drosophila* homolog of PGC-1α, and flies overexpressing *srl* in the muscle under both exercised and unexercised conditions.

**Figure 1:**
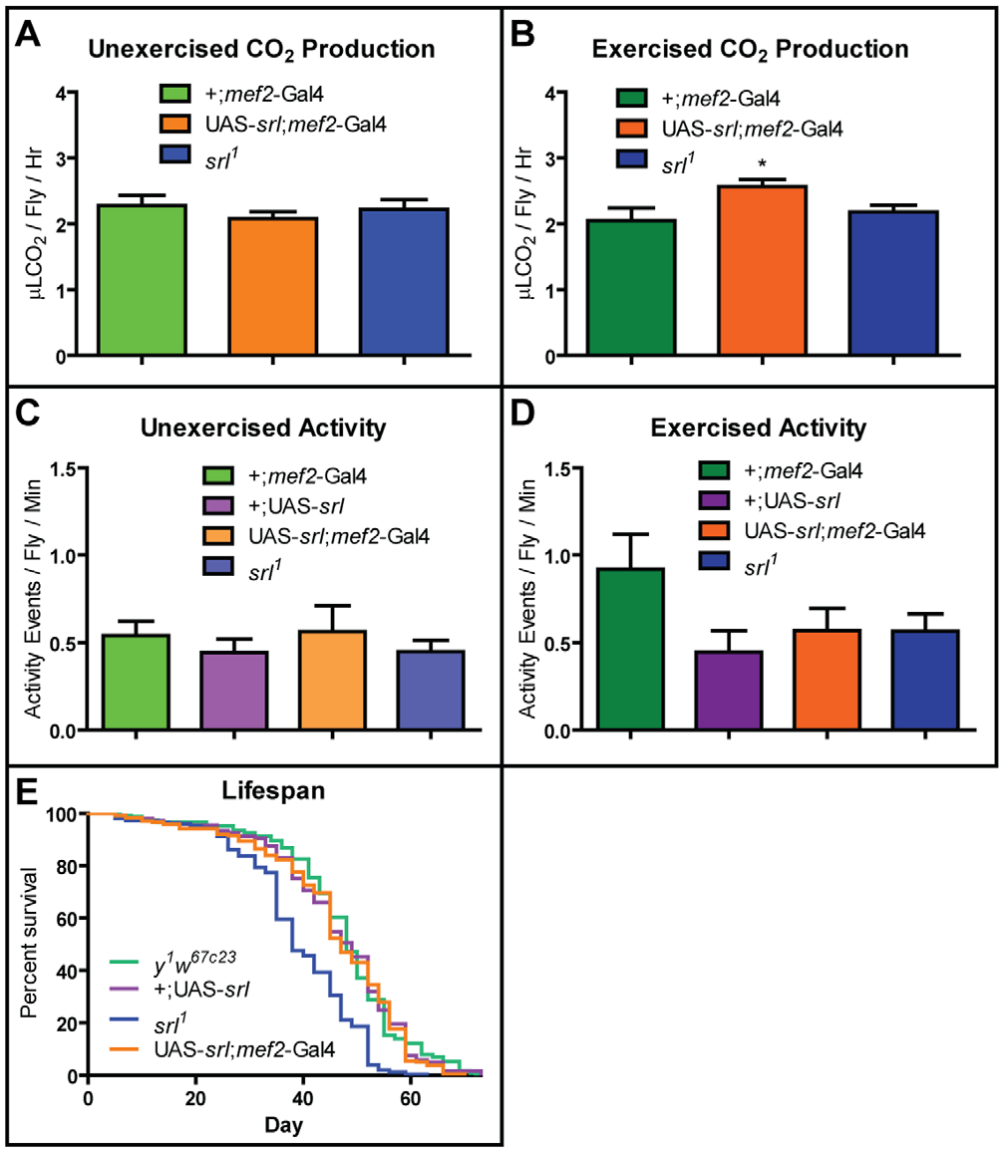
Exercise and *srl* expression in muscle act additively to increase survival and CO_2_ production during treatment. CO_2_ emission rates of 28 day old *srl^1^*, +;*mef2*-Gal4 and UAS-*srl*;*mef2*-Gal4 (**A**) unexercised and (**B**) exercised flies following a three-week exercise regimen. Exercised flies with muscle-specific *spargel* overexpression emit an increased volume of CO_2_ compared with both unexercised *spargel* overexpressing control flies and exercised +;*mef2-*Gal4 flies (*t*-test: p = 0.0217 and p = 0.0283 respectively). Activity levels of 27–29 day old +;*mef2*-Gal4, +;UAS-*srl*, UAS-*srl*;*mef2*-Gal4 and *srl^1^* (**C**) unexercised and (**D**) exercised flies. Exercised flies have statistically similar activity levels to unexercised controls (one-way ANOVA: p = 0.1388). Within each treatment, genotype has no significant effect on activity (one-way ANOVA: p = 0.7563 for unexercised and p = 0.1146 for exercised). Lifespans of female *y^1^w^67c23^*, +;UAS-*srl*, *srl^1^*, and UAS-*srl*;*mef2*-Gal4 flies (**E**). *srl^1^* flies show a significantly a shorter lifespan (**F**) than control flies (log-rank (Mantel-Cox) test: p<0.0001 for all genotype comparisons).

In 25-day old unexercised flies, we detected no significant difference in CO_2_ production when *srl* expression was altered (Figure 1A). In 25-day old flies following three weeks of exercise training, we also found no significant difference in CO_2_ production between *srl^1^* flies and controls (Figure 1B). However, flies overexpressing *srl* in muscle showed a significant increase in CO_2_ production following exercise as compared to controls (Figure 1B; *t*-test: p = 0.0283), or as compared to unexercised overexpressers (compare Figure 1A,1B; *t*-test: p = 0.0217). Thus, neither exercise nor muscle-specific *srl* overexpression was sufficient to increase whole-fly respiration, but the two interventions together produced a 25% increase in whole-fly respiration rate over controls.

### Locomotor Activity

Mammalian studies into the role of PGC-1α in basal locomotor activity have yielded different results under varying experimental procedures. PGC-1α null female mice showed no change in general activity when observed over a two-day time span while PGC-1α null male mice displayed reduced activity when observed for one hour [13]. In a different study, PGC-1α null male mice were found to be hyperactive when observed over a three day time span, likely as a result of lesions in the striatum, a brain area that plays a major role in motor coordination [19]. We find no significant difference in basal activity level in either unexercised (Figure 1C) or exercised (Figure 1D) *srl^1^* flies or muscle-specific *srl* overexpressers (one-way ANOVA followed by Bonferroni posttests: p>0.05 for all comparisons), indicating that *srl* does not significantly alter basal locomotor activity in *Drosophila*.

### Longevity

Lifelong exercise in mammals is known to extend mean lifespan without impacting maximal lifespan [20], [21]. While we find no significant change in the survival of muscle-specific *srl* overexpressers as compared to controls, the *srl^1^* survival curve was significantly shorter than the curves of both *y^1^w^67c23^* and +;UAS-*srl* control flies (Figure 1E; log-rank (Mantel-Cox) test: p<0.0001 for both genotype comparisons), indicating a decreased lifespan in *srl^1^* mutant flies.

### Negative Geotaxis

Endurance training by repetitive induction of a negative geotaxis behavior, as in the *Drosophila* protocol used here, is known to produce an improvement in climbing speed as reflected in the average height reached during a defined time allotted for climbing [1]. Therefore, we performed longitudinal, daily measurements of climbing speed on cohorts of control, *srl* mutant, and *srl*-overexpressing flies, under both exercised and unexercised conditions.

**Figure 2:**
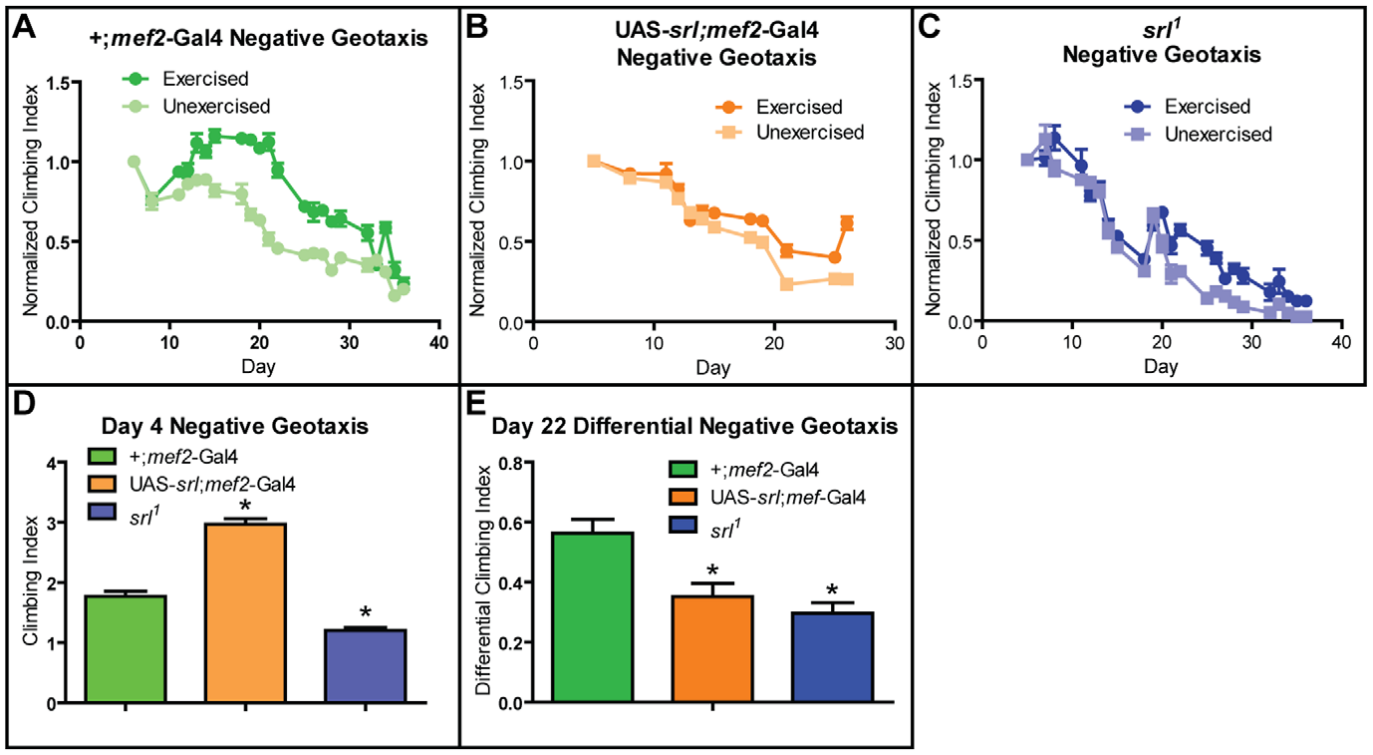
*srl* overexpression is sufficient to improve negative geotaxis. RING assays across ages in (**A**) +;*mef2*-Gal4, (**B**) UAS-*srl*;*mef2*-Gal4, and (**C**) *srl^1^* flies. Exercise-trained flies of all genotypes display improved negative geotaxis ability compared to age-matched unexercised siblings across ages (multivariate regression, treatment-by-age: *srl^1^* mutant and +;*mef2*-Gal4: p<0.001, UAS-*srl*;*mef2*-Gal4: p<0.01). (**D**) Prior to exercise treatment *srl^1^* flies exhibit decreased negative geotaxis ability while UAS-*srl*;*mef2*-Gal4 flies exhibit increased negative geotaxis ability compared to +;*mef2*-Gal4 controls (two-way ANOVA: p<0.001 for both genotype comparisons). (**E**) Relative improvement in climbing index following exercise training, normalized to starting levels for each genotype. Both *srl^1^* and UAS-*srl*;*mef2*-Gal4 flies respond with a significantly smaller increase than WT flies (two-way ANOVA: p<0.001 for both genotype comparisons).

All three genotypes showed a tendency to decline in speed with age (Figure 2A–C), as has been previously reported for a variety of genotypes [1], [22]. However, in all three genotypes, normalized climbing height across time was higher in exercised cohorts than in age-matched, genetically identical, unexercised siblings (Figure 2A–C; multivariate regression, treatment-by-age: *srl^1^* mutant and +;*mef2*-Gal4: p<0.001, UAS-*srl*;*mef2*-Gal4: p<0.01). When unnormalized climbing ability was compared in the same cohorts prior to training at four days of age, flies overexpressing *srl* in muscle displayed significantly higher climbing speed, while *srl^1^* mutant flies were significantly impaired in baseline climbing speed (Figure 2D; *t*-test: p<0.001 for both genotype comparisons). The improvement in exercised climbing speed, relative to unexercised siblings after training at 22 days of life was quantified by expressing the difference between the climbing index of exercised and unexercised flies (Figure 2E). This shows that both a reduction and an increase in *srl* expression decrease the relative change during treatment (*t*-test: p<0.001 for both genotype comparisons).

Taken together, these data indicate that levels of *srl* expression in muscle play an important role in determining climbing speed acutely. However, regardless of *srl* expression levels, exercise treatment still produces some degree of improvement, suggesting that exercise acts through both *srl*-dependent and *srl*-independent pathways.

### Exhaustion

An effect of long-term voluntary exercise in vertebrates is an increase in time to exhaustion when forced to exercise [23]. This parameter has not previously been examined in flies. In order to obtain a quantitative estimate of exhaustion resistance, we continuously induced running on the Power Tower over an extended period of time. We measured both the time at which 50% of flies stopped climbing entirely and the speed at which the flies climbed while undergoing continuous exercise induction.

**Figure 3:**
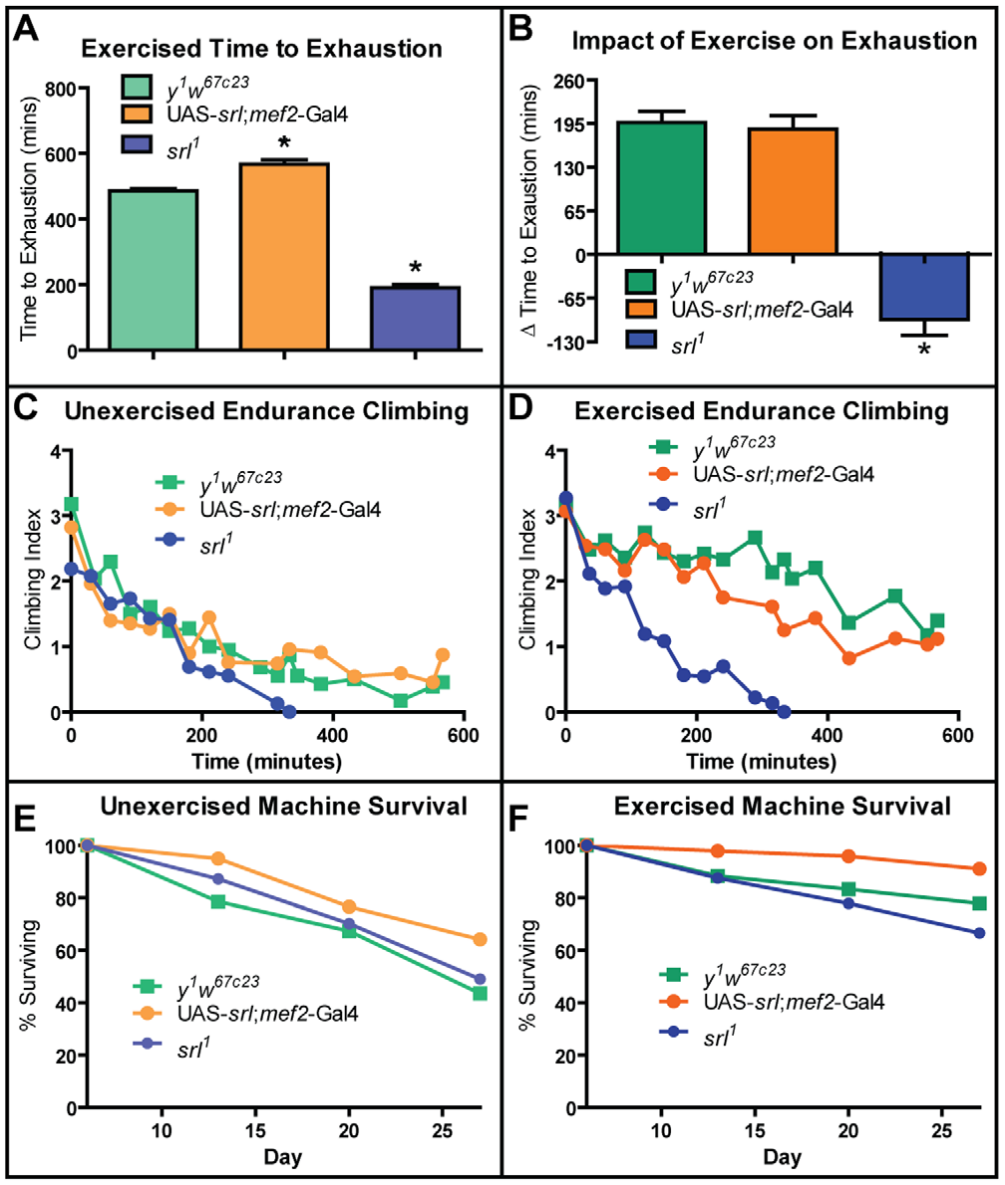
*srl* is required, but not sufficient, for exercise-induced improvement to endurance capacity. (**A**) Time to exhaustion assays conducted on *y^1^w^67c23^*, UAS-*srl*;*mef2*-Gal4, and *srl^1^* flies. Flies overexpressing *srl* in the muscle increase time to exhaustion as compared to *y^1^w^67c23^* control flies (*t*-test: p = 0.0031). (**B**) Change in time to exhaustion in exercised flies as compared to unexercised flies of the same genotype. Wild-type and *srl* overexpressing flies increase time to exhaustion as a result of exercise (*t*-test, exercised and unexercised flies: p = 0.0001 and 0.0004, respectively for each genotype) to the same extent, while *srl^1^* mutant flies in fact decrease time to exhaustion after exercise training (*t*-test, exercised and unexercised *srl^1^*: p = 0.0044, exercised *y^1^w^67c23^* and exercised *srl^1^*: p = 0.0005). (**C**) Endurance climbing assays conducted on 25 day-old *y^1^w^67c23^*, UAS-*srl*;*mef2*-Gal4, and *srl^1^* flies. Unexercised *srl^1^* display a decrease in climbing speed compared to unexercised *y^1^w^67c23^* (multivariate regression, genotype effect: p = 0.0011), while unexercised *srl* overexpressers do not statistically differ from unexercised *y^1^w^67c23^* flies. (**D**) After training, both exercised *y^1^w^67c23^* and exercised UAS-*srl*;*mef2*-Gal4 flies show increased climbing speed when compared to unexercised control flies (multivariate regression, genotype effect: p = 0.0002 and p<0.0001, respectively), though *srl^1^* flies show no significant improvement (multivariate regression, genotype effect: p = 0.817). Survival rates during treatment of (**E**) unexercised and (**F**) exercised *y^1^w^67c23^*, UAS-*srl*;*mef2*-Gal4, and *srl^1^* flies. Unexercised flies were placed on the training machine but not allowed to run. Survival during treatment is statistically improved by exercise in all three genotypes (multivariate regression; treatment-by-age, all p values<0.012). Exercised UAS-*srl*;*mef2*-Gal4 flies also show increased survival compared to exercised *y^1^w^67c23^* flies (multivariate regression, genotype effect, p = 0.0027).

Among cohorts of unexercised flies, overexpression of *srl* in the muscle resulted in increased time to exhaustion (Figure 3A, *t*-test: p = 0.0031). Exercised wild-type and muscle-specific *srl* overexpressing flies both increased time to exhaustion relative to unexercised controls, while *srl^1^* mutant flies actually decreased time to exhaustion as a result of exercise (Figure 3B, *t*-test: p = 0.0005).

Among cohorts of unexercised flies, there was no difference in the endurance climbing speed between control flies and flies overexpressing *srl* in muscle (Figure 3C). However, unexercised *srl^1^* mutant flies showed a statistically significant tendency toward reduced climbing speed (Figure 3C; multivariate regression, genotype-by-time effect: p = 0.0096). In age-matched cohorts previously subjected to endurance training, *srl^1^* mutant flies climbed substantially slower than either control or overexpressing flies (Figure 3D; multivariate regression, genotype-by-time effect: p<0.0001 for both comparisons).

In comparisons between exercised and unexercised flies of the same genotype, both control flies and *srl* overexpressing flies that have undergone exercise training display an approximately two-fold improvement in climbing index following 240 minutes of continuous exercise (Figure 3C,D; *t*-test: p = 0.0183 and p = 0.0209, respectively). In contrast, *srl^1^* flies display no significant improvement when exercised (Figure 3C,D; *t*-test: p = 0.817).

These data indicate that overexpression of *srl* in muscle provides a beneficial resistance to exhaustion in unexercised flies. Reduction in *srl* expression, on the other hand, prevents the exercised-induced increase in time to exhaustion to the extent that exercise actually becomes harmful: exercised *srl^1^* mutant flies become exhausted more quickly than unexercised *srl^1^* mutant flies. These data also indicate that normal levels of *srl* expression are necessary both to maintain a wild-type level of climbing speed and to improve climbing speed during endurance training. However, overexpression of *srl* in muscle provides no additional benefit to exercise-induced increase of climbing speed.

### Survival during treatment

During a standard three-week endurance training protocol, we have previously observed an increase in the death rate of flies during training. The mechanism for this increase in mortality is not fully clear. However, it is specific to the training period and has no effect on post-trained survival (Sujkowski et al., submitted).

To determine whether exercise or *srl* expression levels altered the survival of flies during endurance training, wild-type, *srl* mutant, and *srl*-overexpressing flies were placed on a ramped, three week training program. Concurrently, flies of the same genotypes were also placed on the training machine but were immobilized and prevented from exercising by the insertion of a sponge stopper to limit space for movement.

Among unexercised flies, muscle-specific overexpression of *srl* provided a modest, but statistically significant protection against death during treatment (Figure 3F; multivariate regression, genotype effect, p = 0.0135). Exercise significantly improved survival of all three genotypes (compare Figure 3E,F; multivariate regression; treatment-by-age, all p values<0.012). Among exercised flies, *srl* overexpressing flies again experienced the greatest protection against death (Figure 3F; genotype-by-age, all p values<0.0292), indicating that *srl* acts to protect against death induced by training conditions.

### Cardiac Stress Resistance

Endurance training provides improvements in cardiac metabolism and function in vertebrates [24] and improves long-term cardiac function in flies, protecting against age-related decrease in cardiac stress resistance [1]. To ascertain whether *srl* expression levels altered the effects of exercise on cardiac performance, we performed external electrical pacing assays on control, *srl* mutant, and *srl*-overexpressing flies, under both exercised and unexercised conditions. We first examined flies at four days of age. Exercised cohorts begin training on the fourth day of age, allowing for measurement of fully developed but untrained flies. We also examined 25 day old flies following the completion of exercise training. In unexercised flies, this day represents a median timepoint of age-related decline in cardiac stress resistance [25], thus allowing for detection of either cardiac improvement or impairment.

**Figure 4:**
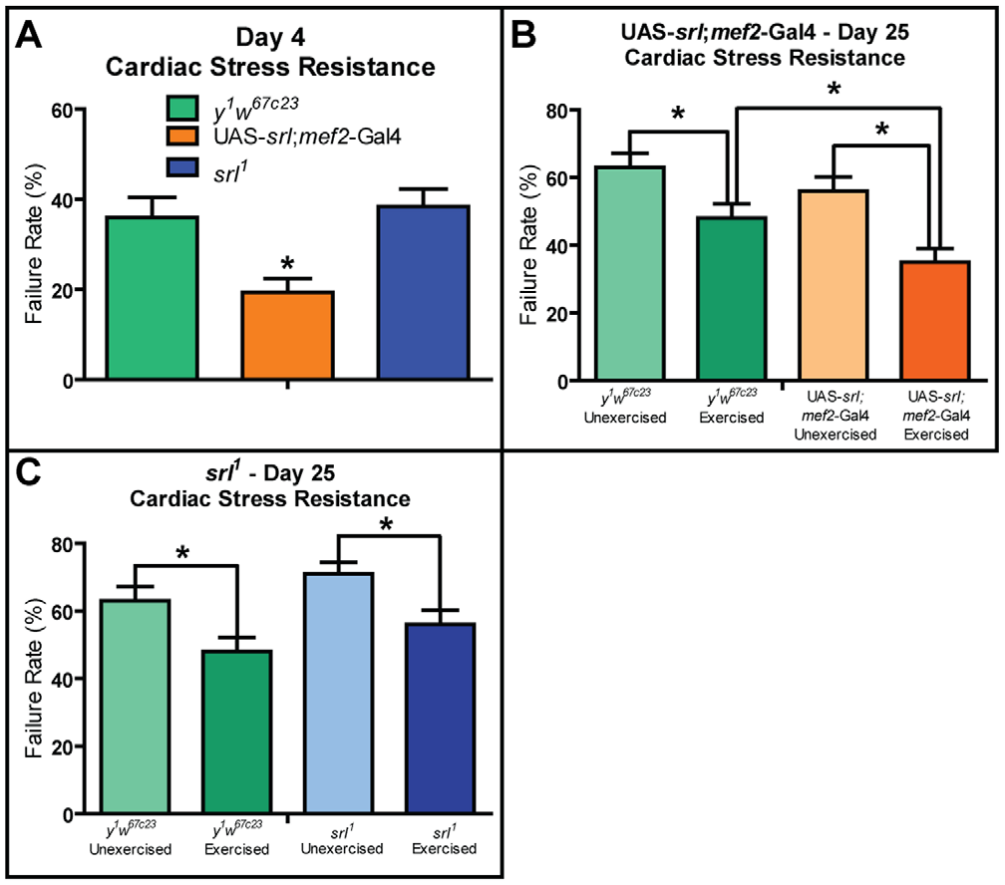
*srl* expression in muscle and heart acts synergistically with exercise to protect from cardiac failure. (**A**) External electrical pacing assays conducted on 4-day old *y^1^w^67c23^*, UAS-*srl*;*mef2*-Gal4, and *srl^1^* flies show reduced stress-induced failure rates in UAS-*srl*;*mef2*-Gal4 flies when compared with *y^1^w^67c23^* controls (F-test, p = 0.0178). Pacing assays conducted on 25 day-old exercised and unexercised (**B**) *y^1^w^67c23^* and UAS-*srl*;*mef2*-Gal4 and (**C**) *y^1^w^67c23^* and *srl^1^* flies show decreased stress-induced failure rates in exercised flies relative to unexercised age-matched siblings in all genotypes (F-test, p = 0.0054, 0.0044, and 0.0394 for control, *srl* overexperssing and *srl* mutant flies respectively). Exercised flies overexpressing *srl* in the muscle also showed a decreased stress-induced failure rate when compared to exercised *y^1^w^67c23^* flies (F-test, p = 0.0430). All *n* were between 56–122.

In four day old flies, prior to the induction of training, *srl^1^* hearts showed similar stress resistance to that of age-matched control flies, whereas flies overexpressing *srl* in muscle, including cardiac muscle, showed a significant improvement in cardiac stress resistance compared to control flies (Figure 4A; F-test, p = 0.0178).

Immediately following completion of a 21 day exercise program, 25 day old exercised cohorts of all genotypes assayed show a reduction in stress-induced failure rate as compared to unexercised flies of the same genotype. (Figure 4B,C; F-test, p = 0.0054, 0.0044, and 0.0394 for control, *srl* overexperssing and *srl* mutant flies respectively). Exercised flies overexpressing *srl* in the muscle also failed at a decreased rate as compared to exercised *y^1^w^67c23^* control flies (Figure 4B, F-test, p = 0.0430). These data suggest that both *srl* and exercise play a cardioprotective role in *Drosophila*, with the greatest decrease in stress-induced failure rate resulting from the overexpression of *srl* in combination with exercise training.

### *srl* transcription

Chronic exercise is known to induce increased transcription [2] and nuclear localization [3] of the vertebrate PGC-1α. We first confirmed that both the Gal4-responsive *srl* overexpression line, and the genomic *srl* mutation, *srl^1^*, effectively modify transcription of *srl* in our hands. We find that UAS-*srl* provides a better than 15-fold increase in *srl* expression (Figure S1A), whereas the *srl^1^* mutant is a hypomorph, with expression about 30% that of wild-type (Figure S1B) as has been previously reported [14]. In contrast to vertebrate PGC-1α, we find no evidence that transcription of *srl* is significantly upregulated by endurance exercise, either in whole flies (Figure S1C), or in the heavily muscle-enriched thoraces (Figure S1D).

## Discussion

### *spargel* Function During Exercise in *Drosophila*

The most well-studied genetic factor in the vertebrate response to endurance training is the transcriptional co-factor PGC-1α. A homolog of PGC-1α, *spargel (srl)*, [14] has been identified in *Drosophila*. Like its vertebrate counterpart, *spargel* upregulates mitochondrial biogenesis and activity [14]. The conservation of the molecular role of *spargel* suggests that its physiological role in response to endurance exercise may also be functionally conserved. Here, we find that *spargel* is necessary for *Drosophila* wild-type exercise performance in several assays. Flies carrying a hypomorphic mutation for *spargel* display reduced survival under endurance exercise conditions, reduced lifespan under standard care conditions, and reduced climbing speed. We also find, however, that *spargel* mutant flies are capable of improving multiple aspects of performance when subjected to training. Taken together, this suggests that *spargel* is required for full exercise capacity, but endurance training can improve performance through mechanisms that are, in part, *spargel*-independent. Alternatively, the reduced expression of the *srl^1^* allele may remain sufficient for some degree of *spargel*-dependent exercise response.

We find that muscle-specific overexpression of *spargel* in *Drosophila* is capable of mimicking some, but not all, of the changes induced by endurance training. Cardiac performance, unexercised time to exhaustion, and initial climbing speed are improved acutely by *spargel* overexpression, while CO_2_ production and time to exhaustion are unaffected. However, flies overexpressing *spargel* also derive additional benefit from endurance training, suggesting again that while *spargel* is an important modulator of exercise physiology in insects, it is not sufficient to account for all the changes induced by training.

Surprisingly, despite the necessity of wild-type *spargel* RNA levels for full exercise capability, we find that *spargel* RNA expression is not significantly upregulated by exercise training. Since PGC-1α is strongly induced by vertebrate exercise [2], this indicates a substantial species difference in regulation. Since PGC-1α is known to change its nuclear localization under exercise conditions [3], it could be that this mechanism predominates in insects. Alternatively, exercise could induce different cofactors in insects that alter the effect of nuclear accumulation of *spargel*. Further work will be necessary to identify the biochemical basis of this difference.

### Conservation of *Drosophila* Exercise Physiology

Since the recent development of *Drosophila* as a model system for exercise physiology, a major point of consideration has been the extent that results from *Drosophila* are likely to be conserved in vertebrates. Here, we demonstrate that the most well-studied genetic factor influencing exercise performance in vertebrates plays a similar role in an insect model.

In both insects and vertebrates, overexpression of either PGC-1α or its homolog is sufficient for increased mitochondrial biogenesis [7], [8], [14]. Similar to our findings, mice overexpressing PGC-1α in muscle show increased respiration capacity when exercised, in addition to increased forced and voluntary running distance [11]. Also comparable to our data, muscle-specific knockout of PGC-1α in rodent models show reduced endurance capacity [26] while PGC-1α null mice show blunted postnatal muscle growth, reduced muscle performance and reduced exercise capacity [13]. PGC-1α manipulation also produces phenotypes that are species-specific. For example, PGC-1α has a vertebrate-specific role in inducing angiogenesis [27] that is inapplicable to insects.

We find that the PGC-1α *Drosophila* homolog *spargel* is a conserved modulator of exercise effects, is required for full exercise capacity in *Drosophila*, and that *spargel* overexpression mimics a subset of changes induced by endurance training. In years to come, the fly model will offer opportunities to study conserved aspects of exercise physiology using the advantages of invertebrate genetic models, such as rapid transgenics, high sample sizes, and inexpensive longitudinal study designs that highlight long-term effects of various manipulations.

## Materials and Methods

### Fly Stocks, Diet and Husbandry

*y^1^w^67c23^*, *CantonS*, *mef2*-Gal4, and *srl^1^* (P{SUPor-P}CG9809^KG08646^) flies were obtained from the Bloomington Stock Center. UAS-*srl* [14] flies were a generous gift from Christian Frei. All UAS and GAL4 insertions were backcrossed into the *y^1^w^67c23^* line at least 10 times. *y^1^w^67c23^*;*mef2*-Gal4/+, *y^1^w^67c23^*;UAS-*srl*/+, and UAS-*srl*/+;*mef2*-Gal4/+ flies are referred to as “+;*mef2*-Gal4,” “+;UAS-*srl*,” and “UAS-*srl*;*mef2*-Gal4” respectively. Standard diet utilized contains 10% yeast, 10% sucrose and 2% agar. During the experimental time course, flies were housed in a 25°C incubator with 50% humitidy and a 12-hour light/dark cycle. Fresh food was provided every other day for the duration of the experiment course. For all experiments flies underwent a three week-long exercise program with controls as described [1]. Flies were exercised using the Power Tower exercise machine, a device which utilizes instinctive negative geotaxis behavior to repetitively induce rapid climbing by lifting and then dropping a platform of flies. Exercised flies were placed on the Power Tower and made to run while unexercised flies were placed on the machine but immobilized with a sponge stopper pushed to the bottom of the vial. Flies were exercised for five days each week over a three week period. The flies were exercised for two hours during the first week, two and a half hours during the second week, and for three hours during the third week.

### Respirometry

CO_2_ production was measured using a flow-through respirometry system. 28 day-old exercised and unexercised +;*mef2*-Gal4, UAS-*srl*;*mef2*-Gal4, and *srl^1^* flies were immobilized 24 hours before measurement by CO_2_ gas and separated into groups of 5 flies per sample. Samples were assayed in random order to evenly distribute variance between measurements. For each measurement, seven samples were transferred into 2 mL glass measurement chambers in a room kept under constant light at 25°C, and one chamber was left empty as a blank reference. Chambers were consecutively flushed for 150 s at a flow rate of 90 ml/min with CO_2_-free, water-saturated room air through an 8-channel MUX flow multiplexer (Sable Systems International, Las Vegas, NV, USA). Flushing of all samples was consecutively repeated 4 times per measurement, resulting in 20 minute intervals during which the chambers were sealed before the 2^nd^, 3^rd^, and 4^th^ flushing. Integrated CO_2_ concentration over time was measured for all samples during the 4th flushing using a Li-7000 CO_2_/H_2_O Analyzer (Sable Systems International, Las Vegas, NV, USA) and used to calculate CO_2_ output over time per fly. All *n* values were between 19–46. Results were analyzed using a two-tailed *t*-test (Prism, GraphPad Software, San Diego, CA, USA).

### Activity

General locomotor activity was assessed using *Drosophila* Activity Monitors (DAM2, Tri-Kinetics, Inc., Waltham, Massachusetts, USA). Exercised and unexercised flies were individually transferred into 5 mm glass tubes containing standard 10% yeast, 10% sucrose and 2% agar fly media. Assays were conducted over three consecutive days from 2:45pm until 6:45pm at a temperature of 24–24.9°C with a relative humidity of 34–40%, during which time the flies were aged between 27 and 29 days. All *n* values = 16. Results were analyzed by one-way ANOVA for overall effect of genotype followed by Bonferroni posttests (Prism, GraphPad Software, San Diego, CA, USA).

### Longevity

240 virgin female flies of each genotype were collected, divided into vials of 20 flies per vial and housed in a 25°C incubator with 50% humidity and a 12-hour light/dark cycle. The flies were transferred every Monday, Wednesday, and Friday to fresh 10% sucrose/10% yeast food and the number of dead flies was recorded. Survival curves were analyzed by long-rank (Mantel-Cox) tests (Prism, GraphPad Software, San Diego, CA, USA).

### Negative Geotaxis Behavior

Negative geotaxis assays were performed in exercised and unexercised flies as described [22] and data was expressed as a normalized climbing index, with the initial average speed of each cohort defined as 1, as described [1]. Non-normalized raw climbing index values were used for figure 2D. To calculate the differential climbing index, unexercised climbing index values from day 22 were subtracted from exercised climbing index values from day 22 for each genotype. All *n* values were between 50 and 120. Results from figures 2A–C were analyzed by multivariate regression and analyzed for the effect of treatment-by-age (JMP, The Statistical Discovery Software, Cary, NC, USA). Results from figures 2D–E were analyzed using a two-tailed *t*-test (Prism, GraphPad Software, San Diego, CA, USA).

### Exhaustion

+;*mef2*-Gal4, UAS-*srl*;*mef2*-Gal4, and *srl^1^* flies were exercised on the power tower using a run-to-exhaustion protocol at 25 days of age. Two cameras were placed on stands in front of the Power Tower exercise machine such that one row of vials was able to be photographed by each camera. White paper was placed behind the vials to create a consistent background. Three vials of 15–20 flies per vial were placed on the power tower for each genotype and treatment. The Power Tower was turned on and the power was adjusted such that the period between drops was 15 s. Pictures were taken 5 seconds after each drop once every 30 min until hour 6. From hour 6 until hour 10, pictures were taken approximately once every hour. Pictures were analyzed by quantifying the number of flies that remained standing on the bottom of the vial as compared to the number of flies that climbed at all up the sides. Flies were considered “exhausted” and the time was recorded once at least 50% of the flies in a vial remained on the bottom of the vial. To assess impact of exercise on exhaustion, the time to exhaustion of exercised flies was subtracted from that of unexercised flies for each genotype. Results were compared using a two-tailed *t*-test (Prism, GraphPad Software, San Diego, CA, USA). Pictures were also analyzed by quantifying negative geotaxis ability as a climbing index as described [1] and results were compared by multivariate regression using time, genotype, and time-by-genotype as construct model effects (JMP, The Statistical Discovery Software, Cary, NC, USA).

### Machine Survival

Dead flies were removed from the study of the course of exercise treatment and the number of remaining flies was charted. Survival rates were averaged over each week and are expressed as a percentage of the initial sample size. Results were compared by multivariate regression to analyze the effect of age-by-genotype (JMP, The Statistical Discovery Software, Cary, NC, USA).

### Electrical Pacing

+;*mef2*-Gal4, UAS-*srl*;*mef2*-Gal4, and *srl^1^* flies were subject to external electrical pacing as described [25]. Following pacing, hearts were visually scored for recovery of normal movement or for “failure,” manifested as either fibrillation or arrest. Heart failure rates were determined for 4 day-old flies and of 25 day-old exercised and unexercised flies. Unexercised *srl^1^* n = 56, all other n values were between 79 and 115. Results were analyzed using a Fisher's exact test for binary measurements (Prism, GraphPad Software, San Diego, CA, USA).

### Real Time RT-PCR

Total RNA was extracted from whole bodies of 12 day-old UAS-*srl*;*tub*-GAL4 and *srl^1^* flies. Total RNA was also extracted from whole bodies and thoraxes of exercised and unexercised *CantonS* flies, as well as untreated *CantonS* flies that were never placed on the machine, with Trizol (Invitrogen, Carlsbad, USA). Whole-body untreated *CantonS* flies were 7 days old, all other *CantonsS* samples were 28 days old. 10–15 flies were used per sample and the resulting RNA was diluted to a common concentration. One-step real time RT-PCR was performed to quantify *srl* expression using the Applied Biosystems StepOnePlus Real-Time PCR System with Power SYBR Green PCR Master Mix (Applied Biosystems, Foster City, USA) according to the following parameters: 1 cycle at 48°C (30 min), 1 cycle at 95°C (10 min), and 40 cycles at 95°C (15 s), 60°C (1 min), followed by melt curve analysis. The average threshold cycles (Ct) for two replicates per sample were used in comparative Ct (ΔΔCt) quantification (Schmittgen and Livak 2008), normalized to expression of the ribosomal gene *rp49*. The following primers were used: *srl* forward: CTCTTGGAGTCCGAGATCCGCAA. *srl* reverse: GGGACCGCGAGCTGATGGTT. *rp49* forward: ACTCAATGGATACTGCCCAAGA. *rp49* reverse: CAAGGTGTCCCACTAATGCATA. Results were analyzed using a two-tailed *t*-test (Prism, GraphPad Software, San Diego, CA, USA).

## Supporting Information

Figure S1 ***srl*** **transcript levels are altered by genotype but not by exercise.** *srl* transcript levels from adult whole-body samples of (**A**) an RU-486 induced *srl* expression construct and (**B**) adult *srl^1^* flies, as compared with adult whole-body samples from *y^1^w^67c23^* control flies (real time RT-PCR: p<0.001). *srl* transcript levels from (**C**) whole-body samples and (**D**) the thoraces of age-matched exercised and unexercised *CantonS* flies, as well as flies never placed on the machine, as determined by real time RT-PCR. Whole-body treatment did not significantly alter expression level.
(TIF)
