# Supplementary figures and images for: The Drosophila PGC-1α Homolog spargel Modulates the Physiological Effects of Endurance Exercise
Source: PLoS One. 2012 Feb 13;7(2):e31633. doi: 10.1371/journal.pone.0031633 (PMC3278454; doi:10.1371/journal.pone.0031633)

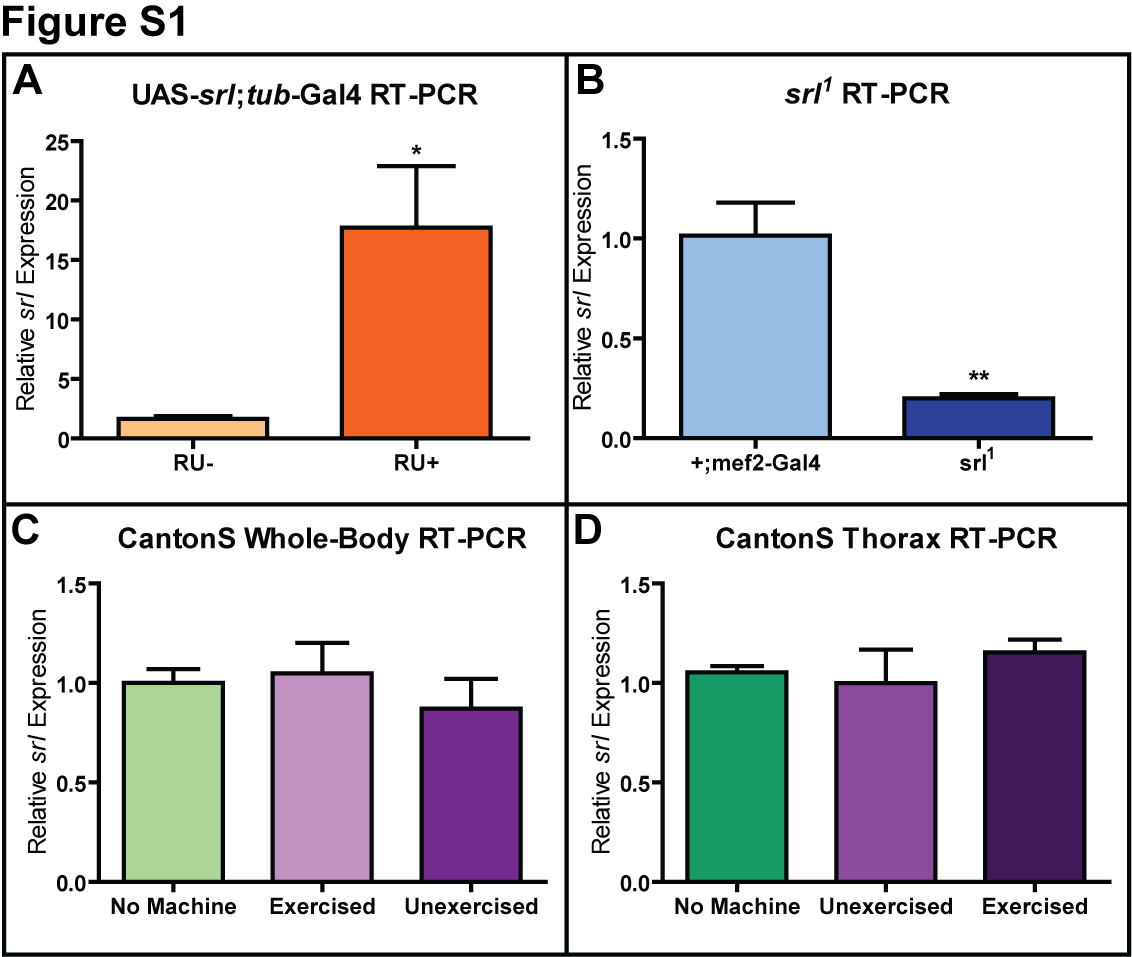

Supplement: Figure S1 — srl transcript levels are altered by genotype but not by exercise. srl transcript levels from adult whole-body samples of (A) an RU-486 induced srl expression construct and (B) adult srl1 flies, as compared with adult whole-body samples from y1w67c23 control flies (real time RT-PCR: p<0.001). srl transcript levels from (C) whole-body samples and (D) the thoraces of age-matched exercised and unexercised CantonS flies, as well as flies never placed on the machine, as determined by real time RT-PCR. Whole-body treatment did not significantly alter expression level. (TIF) [file pone.0031633.s001.tif]
